# Health screenings administered during the domestic medical examination of refugees and other eligible immigrants in nine US states, 2014–2016: A cross-sectional analysis

**DOI:** 10.1371/journal.pmed.1003065

**Published:** 2020-03-31

**Authors:** Clelia Pezzi, Deborah Lee, Gayathri S. Kumar, Breanna Kawasaki, Lori Kennedy, Jenny Aguirre, Melissa Titus, Rebecca Ford, Blain Mamo, Kailey Urban, Stephen Hughes, Colleen Payton, Kevin Scott, Jessica Montour, Emily S. Jentes

**Affiliations:** 1 Centers for Disease Control and Prevention, Division of Global Migration and Quarantine, Immigrant, Refugee, and Migrant Health Branch, Atlanta, Georgia, United States of America; 2 Colorado Department of Public Health and Environment, Disease Control and Environmental Epidemiology Division, Refugee Health Program, Denver, Colorado, United States of America; 3 Illinois Department of Public Health, Refugee Health Program, Chicago, Illinois, United States of America; 4 Marion County Public Health Department, Indianapolis, Indiana, United States of America; 5 University of Louisville Division of Infectious Diseases, Louisville, Kentucky, United States of America; 6 Minnesota Department of Health, Saint Paul, Minnesota, United States of America; 7 Bureau of Tuberculosis Control, New York State Department of Health, Albany, New York, United States of America; 8 Department of Family & Community Medicine, Thomas Jefferson University, Philadelphia, Pennsylvania, United States of America; 9 Texas Department of State Health Services, Austin, Texas, United States of America; Johns Hopkins University Bloomberg School of Public Health, UNITED STATES

## Abstract

**Background:**

Refugees and other select visa holders are recommended to receive a domestic medical examination within 90 days after arrival to the United States. Limited data have been published on the coverage of screenings offered during this examination across multiple resettlement states, preventing evaluation of this voluntary program’s potential impact on postarrival refugee health. This analysis sought to calculate and compare screening proportions among refugees and other eligible populations to assess the domestic medical examination’s impact on screening coverage resulting from this examination.

**Methods and findings:**

We conducted a cross-sectional analysis to summarize and compare domestic medical examination data from January 2014 to December 2016 from persons receiving a domestic medical examination in seven states (California, Colorado, Minnesota, New York, Kentucky, Illinois, and Texas); one county (Marion County, Indiana); and one academic medical center in Philadelphia, Pennsylvania. We analyzed screening coverage by sex, age, nationality, and country of last residence of persons and compared the proportions of persons receiving recommended screenings by those characteristics. We received data on disease screenings for 105,541 individuals who received a domestic medical examination; 47% were female and 51.5% were between the ages of 18 and 44. The proportions of people undergoing screening tests for infectious diseases were high, including for tuberculosis (91.6% screened), hepatitis B (95.8% screened), and human immunodeficiency virus (HIV; 80.3% screened). Screening rates for other health conditions were lower, including mental health (36.8% screened). The main limitation of our analysis was reliance on data that were collected primarily for programmatic rather than surveillance purposes.

**Conclusions:**

In this analysis, we observed high rates of screening coverage for tuberculosis, hepatitis B, and HIV during the domestic medical examination and lower screening coverage for mental health. This analysis provided evidence that the domestic medical examination is an opportunity to ensure newly arrived refugees and other eligible populations receive recommended health screenings and are connected to the US healthcare system. We also identified knowledge gaps on how screenings are conducted for some conditions, notably mental health, identifying directions for future research.

## Abstract 2

In a cross-sectional analysis, Clelia Pezzi and colleagues assess selected health screenings administered during the domestic medical examination of refugees and other eligible immigrants in nine US states, 2014–2016.

## Author summary

**Why was this analysis done?:**

- Newly arrived refugees, special immigrant visa holders, asylees, Cuban/Haitian entrants, and other select visa types are eligible for a domestic medical examination conducted shortly after they arrive in the US. The purpose of this examination is to identify health conditions requiring further treatment and to connect these populations to the US healthcare system.
- Several investigations have reported on health conditions identified during the domestic medical examination, but none have assessed the impact of this examination on recommended health screening coverage for these populations.
- We sought to better understand how the domestic medical examination impacts screening coverage among eligible populations in selected jurisdictions in the US.

**What did the authors do and find?:**

- We collected data from domestic screening examinations conducted between 2014 and 2016 from nine sites across the US, including basic demographic data and specific health screening tests administered during the examination.
- We analyzed proportions of people screened for tuberculosis, human immunodeficiency virus (HIV), hepatitis B and C, various parasites, blood lead level, and mental health conditions. We also described screening coverage by age, sex, visa type, examination year, nationality, and country of last residence.
- Among the 105,541 persons who received a domestic screening examination, most persons were screened for tuberculosis (91.6%), hepatitis B (95.8%), and HIV (80.3%). Mental health screening was lower (36.8%).

**What do these findings mean?:**

- The domestic medical examination is an effective opportunity to screen eligible newly arrived populations for several health conditions, particularly tuberculosis, HIV, and hepatitis B, but may not be as effective an opportunity for mental health screening.
- The high screening coverage during the domestic screening examination ensures that refugees and other eligible populations are healthily integrated into receiving communities.
- Future analyses could be conducted to better understand why screening rates for mental health are low during the domestic medical examination and how effectively the examination links individuals to the US healthcare system.
- The main limitation of our analysis was our use of nonstandard data collected primarily for programmatic rather than surveillance purposes.

## Introduction

Between 2014 and 2016, the US welcomed an annual average of 75,000 refugees, 9,900 Special Immigrant Visa holders (SIVHs), and 23,000 persons granted asylum (asylees) [1]. Before US resettlement, these populations may have spent years without access to routine, preventive healthcare [2]. To address healthcare gaps and ensure healthy resettlement in the US, the immigration process includes multiple health screening exams and interventions [3].

Under section 325 of the Public Health Service Act (42 USC 252) and sections 212 and 232 of the Immigration and Nationality Act (8 USC 1182, 1222), refugees, SIVHs, and derivative asylees (persons applying outside of the US) are required to undergo a medical exam before admission to the US to detect the presence of inadmissible health conditions. These medical exams are conducted by physicians contracted by the US Department of State and are performed according to US Centers for Disease Control and Prevention (CDC) *Technical Instructions for the Medical Examination of Aliens* [3]. In addition, US-bound refugees (but not other visa types) are eligible for overseas health interventions such as predeparture screening, the voluntary Vaccination Program for US-bound Refugees, and the presumptive parasite treatment program [3]. Asylees granted their status after US arrival do not receive overseas screening or interventions [4].

After US arrival or certification, refugees, SIVHs, asylees, Cuban/Haitian entrants (parolees), Amerasians (a visa status available to children born in Vietnam of US citizen fathers after January 1, 1962, and before January 1, 1976, and their eligible dependents), and certified victims of human trafficking are eligible for benefits, including a recommended medical screening examination that addresses health conditions diagnosed overseas, identifies additional health concerns, and connects the individuals with the US healthcare system [3,5]. CDC provides *Guidelines for the U*.*S*. *Domestic Medical Examination for Newly Arriving Refugees*, but state or local refugee health programs independently coordinate the examination, and these guidelines may be adapted for local use [6,7]. The comprehensive examination includes a medical history and physical examination, screening for select communicable diseases, nutritional status assessment, blood lead screening for children <17 years old, and immunization administration according to US schedules [7].

Examinations and interventions provided during the initial resettlement period ensure that refugees and other eligible populations are healthily integrated into receiving communities and are ready to begin work and school. Further, the domestic examination is usually the first contact the individual has with the US healthcare system and possibly the first time he/she is screened for certain health conditions or receives preventive healthcare [8,9].

Assessing the impact of the domestic examination on the recipient as well as on the receiving community has been challenging. Data from these examinations are not routinely collected at the national level, and state capacity to collect and analyze these data varies. In a review of the literature, we found that most analyses based on domestic medical examination data occur at the clinic, state, or local levels [10] and may not represent all recipients of refugee health program services, given the heterogeneity of these populations. Where domestic examination data are pooled across states or sites, analyses are generally limited to a particular condition (e.g., hepatitis B, lead) or population (e.g., children) [11–14]. Finally, we identified few analyses that reported the proportion of persons screened by condition during the domestic medical examination [10–12], which, to our knowledge, prevents assessment of the impact of the domestic examination on screening coverage. Accordingly, we conducted, to our knowledge, a novel analysis to assess screening coverage during the domestic medical examination on screening coverage for various conditions by calculating and comparing screening proportions among refugees and other visa holders by select demographic characteristics.

## Methods

We conducted a retrospective, cross-sectional analysis of data derived from domestic medical examinations administered to refugees, SIVHs, asylees, parolees, and victims of trafficking between January 1, 2014, and December 31, 2016, in seven states (California, Colorado, Minnesota, New York, Kentucky, Illinois, and Texas); one county (Marion County, Indiana); and one academic medical center in Philadelphia, Pennsylvania. These sites provided their data as part of the CDC-funded CK12-1205 Strengthening Surveillance for Diseases among Newly Arrived Immigrants and Refugees nonresearch cooperative agreement administered by CDC. At least one representative of each site participated in the data collection and analysis of data. Although this is a convenience sample and not generalizable to all refugees in the US, the data set includes data from three states (California, Texas, and New York) with the highest volume of refugee, asylee, and SIVH arrivals between 2014 and 2016 and represents 28% of all refugee, asylee, and SIVH arrivals to the US during the same period. As part of a larger data collection effort, this analysis was not guided by a specific prospective analysis plan. However, the variables collected for analysis were outlined in a protocol used for ethical determination and shared with site partners contributing data. After data collection, we assessed the quality and availability of our data and identified appropriate analyses, including this descriptive analysis to assess screening coverage. This activity received a nonresearch determination from a CDC human subjects adviser and was exempt from further ethics review.

We suggested the variables and formats to compile the data to the nine participating site data partners. If partners could not provide those variables, they shared comparable raw data with explanations for missing variables or data. We then recoded the raw data for inclusion in the combined data set. Most data were collected from the state or local refugee health program databases, but some partners obtained data from other state or local programs (e.g., tuberculosis [TB] control or lead poisoning) because those data were not in the refugee program database. Some sites’ restrictions on sharing personally identifying information prevented them from sharing birth and arrival dates; instead, they provided age at arrival and arrival year as proxy variables. Sites either provided nationality and country of last residence or shared a unique identifier we used to link to the US Department of State’s Worldwide Refugee Admissions Processing System (78.1% of records linked) and CDC’s Electronic Disease Notification system (74.0% linked) to obtain these two variables. Nationality or country of last residence were left blank or imputed from country of birth for records that did not match. Other than the unique identifier and birth date, we did not collect or store personally identifying information. Data were transmitted to CDC via secure file transfer and stored behind CDC’s firewall and physical security measures; access to raw data was limited to a few CDC refugee health program staff.

Demographic variables were age (in years), sex, arrival year, visa status, nationality, and country of last residence (generally the country of asylum for refugees). For our outcome of interest, screening status, we created a bivariate variable of “screened,” which included all persons a site reported to be screened, regardless of outcome, and “not screened,” which included persons who were not screened; screening status was known for all persons. We examined results for conditions with screening recommended by CDC’s *Guidelines for the U*.*S*. *Domestic Medical Examination for Newly Arriving Refugees* (Table 1): TB (using interferon gamma release assays [IGRAs] or tuberculin skin tests [TST]; screening tests do not distinguish between latent TB infection [LTBI] and TB disease), hepatitis B (results from hepatitis B surface antigen [HBsAg] and hepatitis B core antibody [anti-HBc] and/or hepatitis B surface antibody [anti-HBs] testing, when available), hepatitis C, malaria, strongyloidiasis, schistosomiasis, other intestinal parasites, syphilis, gonorrhea, chlamydia, human immunodeficiency virus (HIV), blood lead levels (among children <17 years old), and mental health. For most conditions, we were unable to collect detailed information on the method of screening used by partners. We assumed the sites conducted screening or testing in accordance with the CDC’s *Guidelines for the U*.*S*. *Domestic Medical Examination for Newly Arriving Refugees* (Table 1).

**Table 1:** Recommended health screenings of refugees and other eligible immigrants during the domestic medical screening examinations conducted in nine selected US sites according to CDC guidelines, 2014–2016^a^.

Health Condition	Populations Recommended for Screening	Screening
**Tuberculosis**	All groups	IGRA and/or TST
**Hepatitis B Virus**	All individuals who were born in or most recently lived in countries in which the rate of chronic hepatitis B virus infection is >2% (intermediate or highly endemic countries) and all individuals from low-endemicity countries if they have hepatitis B virus risk factors or are pregnant	HBsAg and anti-HBc (optional) and/or anti-HBs (optional)
**Hepatitis C Virus**	High-risk groups, including people who have ever injected illegal drugs, have body art (tattoos, piercings), are human immunodeficiency virus positive, have a history of multiple sex partners or sexually transmitted infections, have a mother positive for hepatitis C virus, received blood products before migration, have a history of chronic hemodialysis, or have other risk factors	Anti-HCV or RIBA or hepatitis C ribonucleic acid PCR
**Malaria**	Sub-Saharan African refugees receiving no presumptive treatment before departure or any person from a malaria-endemic country with signs or symptoms of infection	Laboratory-confirmed via microscopy or rapid diagnostic test
***Strongyloides***	Individuals without complete predeparture treatment	Laboratory-confirmed microscopy or serology test
***Schistosoma***	Individuals coming from sub-Saharan Africa without complete predeparture treatment (most refugees are treated overseas)	Laboratory-confirmed microscopy or serology test
**Intestinal Parasites**	Individuals with no predeparture treatment (most refugees from Middle East, South and Southeast Asia, and Africa are treated overseas)	Laboratory-confirmed stool ova and parasite test
**Syphilis**	If no overseas testing documentation: (1) all persons ≥15 years of age, regardless of the overseas results, and (2) refugees <15 years if they are sexually active/have a history of sexual abuse, have a mother who tests positive, are from a country endemic for other treponemal subspecies	Non-treponemal test (Venereal Disease Research Laboratory or rapid plasma reagin)
**Gonorrhea**	Anyone with symptoms or leukocyte esterase positive on urine sample and women and children with history or risk of sexual assault	Laboratory-confirmed testing (not specified)
**Chlamydia**	Sexually active women <25 years old, women >25 years old with risk factors, women or children with sexual assault history or risk, anyone with symptoms or are leukocyte esterase positive on urine sample	Laboratory-confirmed testing (not specified)
**Human Immunodeficiency Virus**	All groups (opt-out screening)	Laboratory-confirmed testing (not specified)
**Blood Lead**	All children 6 months to 16 years old	Blood lead level from capillary or venous specimen (venous preferred)
**Mental Health**	Individuals >16 years old should be screened for symptoms of major depression and posttraumatic stress disorder	RHS-15 or diagnosis or notation of mental condition including posttraumatic stress disorder, depression, anxiety, adjustment disorder, alcohol or substance abuse, and/or generalized anxiety disorder

^a^This table reflects CDC’s *Guidelines for the U*.*S*. *Domestic Medical Examination for Newly Arriving Refugees* in place during the period of this analysis (2014–2016). Changes to the guidelines and broader implementation of overseas intervention programs occurred during and after the data collection for this analysis, which may have affected domestic screening for some groups. Current guidelines can be found at https://www.cdc.gov/immigrantrefugeehealth/guidelines/domestic/domestic-guidelines.html

Abbreviations: anti-HBc, hepatitis B core antibody; anti-HBs, hepatitis B surface antibody; anti-HCV, hepatitis C antibody; CDC, Centers for Disease Control and Prevention; HBsAg, hepatitis B surface antigen; IGRA, interferon gamma release assay; PCR, polymerase chain reaction; RHS, Refugee Health Screener; RIBA, recombinant immunoblot assay; TST, tuberculin skin test

We used SAS 9.4 (SAS Institute, Cary, NC) to combine, clean, and standardize data from each site, resulting in an initial data set of 107,162 records meeting our inclusion criteria. A unique identifier was available for six sites (*n* = 90,209, 84.2%); data sharing laws in three sites (*n* = 16,953, 15.8%) prevented sharing of unique identifiers. We used the unique identifier to remove 1,621 (1.8%) duplicate records. Most duplicate records (*n* = 1,430, 88.2%) were from one site and related to a reporting error unlikely to have been repeated in other sites, in which the corrected rate of duplicate records was 0.2%. Therefore, we estimate the potential number of duplicate records remaining in the portion of data set without unique identifiers to be low (approximately 0.2%). We presented data individually for the top 10 countries of nationality and last residence by arrival volume and grouped the remaining countries into an “other” category. Denominators for each condition consisted of the total number of persons for whom screening status (e.g., screened or not screened) was available, which resulted in different denominators by condition. For each condition, we excluded data from sites that were only able to provide positive testing results without providing data on how many people were screened for the condition or who did not routinely screen or report data on a specific condition. The denominators used to calculate proportions screened are reported in “Total” column of each table. We calculated frequencies and percentages and their 95% confidence intervals (CIs) for demographic and screening variables and cross-tabulations for the number and proportion of persons screened with valid results for each condition by age, sex, visa type, examination year, countries of nationality, and last residence and for TB only, the screening test type. We used χ^2^ and Fischer exact tests to compare proportions screened by age, sex, visa type, exam year, nationality, and country of last residence. We considered differences in proportions statistically significant at the 0.05 alpha level and examined 95% CIs to assess stratum-specific differences in screening by characteristic. This analysis is reported per the Strengthening the Reporting of Observational Studies in Epidemiology (STROBE) guideline (S1 STROBE Checklist).

## Results

Our analysis data set included 105,541 refugees and other eligible persons who received a domestic medical examination between January 1, 2014, and December 31, 2016, in one of the nine sites. Characteristics of the data set are reported by visa status in Table 2. All nine sites provided screening data for TB, hepatitis B, HIV, and hepatitis C, resulting in a denominator of 105,541 persons screened for those conditions (Table 3). Eight sites provided screening data for malaria, schistosomiasis, strongyloidiasis, intestinal parasites, and sexually transmitted infections (STIs), resulting in a denominator of 100,677 persons screened for these infections (Tables 4 and 5). All nine sites provided blood lead screening data for children <17 years old, which is the age recommendation for lead screening in CDC’s *Guidelines for the U*.*S*. *Domestic Medical Examination for Newly Arriving Refugees* (lead screening denominator: 35,118, Table 6). Finally, five sites provided mental health screening data for 27,712 persons (Table 6). Four sites did not share mental health screening data because they either did not conduct mental health screening at the time of the domestic screening examination or did not retain mental health screening information in the data sources used for this analysis.

**Table 2:** Demographic characteristics of persons receiving a domestic medical examination by visa status in nine US sites, 2014–2016.

Characteristics	Visa Status											
	Total		Asylee		Parolee		Refugees		Special Immigrant Visa Holders		Other^a^	
	*N*	Col%	*N*	Col%	*N*	Col%	*N*	Col%	*N*	Col%	*N*	Col%
**Total (Row %)**	105,451	100	4,044	3.8	14,915	14.1	74,025	70.2	11,801	11.2	666	0.6
**Mean Age, Years (SD)**	25.8 (17.1)		28.1 (16.1)		33.2 (13.0)		25.1 (17.8)		19.9 (14.4)		26.2 (16.1)	
**Age (Years)**^b^												
**<5**	12,096	11.5	213	5.3	358	2.4	9,023	12.2	2,447	20.7	55	8.3
**5–17**	24,614	23.3	930	23.0	989	6.6	19,882	26.9	2,651	22.5	162	24.3
**18–44**	54,255	51.5	2,267	56.1	10,906	73.1	34,506	46.6	6,215	52.7	361	54.2
**45–64**	11,714	11.1	552	13.7	2,411	16.2	8,258	11.2	417	3.5	76	11.4
**65+**	2,762	2.6	82	2.0	251	1.7	2,347	3.2	70	0.6	12	1.8
**Examination Year**												
**2014**	34,548	32.8	1,818	45.0	3,408	22.9	25,410	34.3	3,673	31.1	239	35.9
**2015**	33,358	31.6	1,240	30.7	6,168	41.4	22,533	30.4	3,190	27.0	227	34.1
**2016**	37,545	35.6	986	24.4	5,339	35.8	26,082	35.2	4,938	41.8	200	30.0
**Sex**^c^												
**Female**	1,933	47.8	1,933	47.8	5,942	39.8	35,977	48.6	5,291	44.8	374	56.2
**Male**	2,111	52.2	2,111	52.2	8,970	60.1	38,036	51.4	6,509	55.2	288	43.2
**Median Days Elapsed between Arrival and Examination (IQR)**	30 (16–53)		57 (40–74)		49 (31–71)		27 (14–47)		27 (14–48)		47 (29–67)	
**Days from Arrival to Exam**												
**<30 Days**	53,070	50.3	595	14.7	3,558	23.9	42,284	57.1	6,450	54.7	183	27.5
**30–90 Days**	48,164	45.7	3,113	77.0	10,173	68.2	29,449	39.8	5,025	42.6	404	60.7
**>90 Days**	3,384	3.2	318	7.9	1,009	6.8	1,770	2.4	216	1.8	71	10.7
**Unknown**	833	0.8	18	0.5	175	1.2	522	0.7	110	0.9	8	1.2
**Nationality**												
**Afghanistan**	11,736	11.1	191	4.7	33	0.2	2,397	3.2	8,977	76.1	138	20.7
**Bhutan**	4,221	4.0	6	0.2	4	0.0	4,201	5.7	4	0.0	6	0.9
**Burma**	17,749	16.8	29	0.7	9	0.1	17,674	23.9	29	0.3	8	1.2
**Democratic Republic of Congo**	6,554	6.2	28	0.7	≤3	0.0	6,497	8.8	25	0.2	≤3	0.5
**Cuba**	15,459	14.7	29	0.7	14,499	97.2	867	1.2	44	0.4	20	3.0
**Iran**	7,179	6.8	367	9.1	37	0.3	6,396	8.6	350	3.0	29	4.4
**Iraq**	16,605	15.8	389	9.6	24	0.2	14,172	19.1	1,963	16.6	57	8.6
**Somalia**	8,474	8.0	110	2.7	≤3	0.0	8,288	11.2	44	0.4	30	4.5
**Syria**	4,258	4.0	82	2.0	≤3	0.0	4,125	5.6	45	0.4	4	0.6
**Ukraine**	1,485	1.4	22	0.5	6	0.0	1,362	1.8	93	0.8	≤3	0.3
**Other**^d^	11,731	11.1	2,791	69.0	298	2.0	8,046	10.9	227	1.9	369	55.4
**Last Residence**												
**Afghanistan**	8,475	8.0	162	4.0	29	0.2	998	1.4	7,153	60.6	133	20.0
**Austria**	5,173	4.9	84	2.1	33	0.2	4,773	6.5	270	2.3	13	2.0
**Cuba**	3,791	3.6	18	0.5	2,997	20.1	741	1.0	24	0.2	11	1.7
**Iraq**	8,781	8.3	131	3.2	15	0.1	7,089	9.6	1,499	12.7	47	7.1
**Jordan**	4,851	4.6	44	1.1	5	0.0	4,743	6.4	55	0.5	4	0.6
**Kenya**	4,361	4.1	19	0.5	≤3	0.0	4,305	5.8	21	0.2	13	2.0
**Malaysia**	11,217	10.6	12	0.3	7	0.1	11,167	15.1	26	0.2	5	0.8
**Nepal**	4,319	4.1	117	2.9	4	0.0	4,188	5.7	4	0.0	6	0.9
**Thailand**	6,363	6.0	5	0.1	≤3	0.0	6,338	8.6	16	0.1	≤3	0.2
**Turkey**	5,586	5.3	45	1.1	≤3	0.0	5,369	7.3	162	1.4	7	1.1
**Other**^e^	42,534	40.3	3,407	84.3	11,816	79.2	24,314	32.9	2,571	21.8	426	64.0

Col% refers to the proportion of persons with characteristics using the total population (in first column) as denominator.

^a^“Other” visa category includes victims of trafficking, unaccompanied minors, and persons with an unspecified visa status.

^b^Excludes 10 individuals missing age.

^c^Excludes 20 individuals missing sex.

^d^Includes persons with the following nationalities: Missing, Algeria, Andorra, Angola, Armenia, Austria, Azerbaijan, Bangladesh, Belarus, Belize, Benin, Botswana, Brazil, Burkina Faso, Burundi, Cambodia, Cameroon, Canada, Central African Republic, Chad, Chile, China, Colombia, Republic of Congo, Costa Rica, Côte d’Ivoire, Cyprus, Djibouti, Dominican Republic, Ecuador, Egypt, El Salvador, Eritrea, Estonia, Ethiopia, Fiji, Gabon, Gaza Strip/West Bank, Georgia, Germany, Ghana, Greece, Grenada, Guatemala, Guinea, Haiti, Honduras, India, Indonesia, Ireland, Israel, Jamaica, Japan, Jordan, Kazakhstan, Kenya, Kuwait, Kyrgyzstan, Latvia, Lebanon, Liberia, Libya, Malawi, Malaysia, Maldives, Mali, Mexico, Moldova, Mongolia, Morocco, Mozambique, Namibia, Nepal, the Netherlands, Nicaragua, Niger, Nigeria, North Korea, Norway, Oman, Pakistan, Peru, Philippines, Poland, Polish, Qatar, Romania, Russia, Rwanda, Saudi Arabia, Senegal, Serbia, Sierra Leone, Solomon Islands, South Africa, South Korea, South Sudan, Spain, Sri Lanka, Sudan, Sweden, Taiwan, Tajikistan, Tanzania, Thailand, Togo, Tunisia, Turkey, Turkmenistan, Uganda, United Arab Emirates, Unknown, Uzbekistan, Venezuela, Vietnam, Yemen, Zambia, and Zimbabwe.

^e^Includes persons with the following countries of last residence: Missing, Albania, Algeria, Armenia, Australia, Azerbaijan, Bahrain, Bangladesh, Belarus, Belize, Bhutan, Botswana, Brazil, Burma, Burundi, Cambodia, Cameroon, Canada, Central African Republic, Chad, China, Colombia, Costa Rica, Côte d’Ivoire, Democratic Republic of Congo, Djibouti, Dominican Republic, Ecuador, Egypt, El Salvador, Eritrea, Ethiopia, France, Gabon, Gaza Strip/West Bank, Germany, Ghana, Guatemala, Guinea, Haiti, Honduras, Hong Kong, India, Indonesia, Iran, Israel, Italy, Kazakhstan, Kuwait, Kyrgyzstan, Latvia, Lebanon, Liberia, Libya, Malawi, Mali, Malta, Mauritania, Mexico, Moldova, Mongolia, Morocco, Mozambique, Myanmar, Namibia, the Netherlands, Nicaragua, Nigeria, North Korea, Oman, Pakistan, Panama, Peru, Philippines, Qatar, Republic of Congo, Romania, Russia, Rwanda, Saudi Arabia, Slovakia, Slovenia, Somalia, South Africa, South Korea, Spain, Sri Lanka, Sudan, Sweden, Syria, Tajikistan, Tanzania, Togo, Trinidad and Tobago, Tunisia, Uganda, Ukraine, United Arab Emirates, United Kingdom, United States, Uzbekistan, Venezuela, Vietnam, Yemen, Zambia, Zimbabwe.

Abbreviations: SD, standard deviation

**Table 3:** Screening coverage of refugees and other eligible immigrants for tuberculosis infection, hepatitis B, HIV, and hepatitis C by demographic characteristics during domestic medical screening examinations conducted in nine US sites, 2014–2016.

Characteristics			Number and Proportion of Persons Screened											
	Total		Tuberculosis Infection			Hepatitis B			HIV			Hepatitis C		
	*N*	Col%	*N*	Row%	χ^2^ 95% CI	*N*	Row%	χ^2^ 95% CI	*N*	Row%	χ^2^ 95% CI	*N*	Row%	χ^2^ 95% CI
**Total**	105,451	100	96,606	91.6	91.4–91.8	101,004	95.8	95.7–95.9	84,687	80.3	80.1–80.6	49,783	47.2	46.9–47.5
**Age (Years)**^a^					<0.001			<0.001			<0.001			<0.001
**<5**	12,096	11.5	7,489	61.9	61.1–62.8	10,402	86	85.4–86.6	7,862	65.0	64.2–65.9	4,433	36.7	35.8–37.5
**5–17**	24,614	23.3	22,628	91.9	91.6–92.3	23,257	94.5	94.2–94.8	18,275	74.3	73.7–74.8	10,635	43.2	42.6–43.8
**18–44**	54,255	51.5	52,445	96.7	96.5–96.8	53,112	97.9	97.8–98.0	46,105	85	84.7–85.3	26,593	49	48.6–49.4
**45–64**	11,714	11.1	11,351	96.9	96.6–97.2	11,508	98.2	98.0–98.5	10,034	85.7	85.0–86.3	6,330	54	53.1–54.9
**65+**	2,762	2.6	2,683	97.1	96.5–97.8	2,715	98.3	97.8–98.8	2,402	87.0	85.7–88.2	1,789	64.8	63.0–66.6
**Examination Year**					<0.001			<0.001			<0.001			<0.001
**2014**	34,548	32.8	31,788	92	91.7–92.3	33,005	95.5	95.3–95.8	27,148	78.6	78.2–79.0	16,689	48.3	47.8–48.8
**2015**	33,358	31.6	30,585	91.7	91.4–92.0	32,294	96.8	96.6–97.0	28,000	83.9	83.5–84.3	15,980	47.9	47.4–48.4
**2016**	37,545	35.6	34,233	91.2	90.9–91.5	35,705	95.1	94.9–95.3	29,539	78.7	78.3–79.1	17,114	45.6	45.1–46.1
**Sex**^b^					<0.001			0.0012			<0.001			<0.001
**Female**	49,517	47	45,211	91.3	91.1–91.6	47,325	95.6	95.4–95.8	39,554	79.9	79.5–80.2	23,767	48	47.6–48.4
**Male**	55,914	53	51,386	91.9	91.7–92.1	53,663	96	95.8–96.1	45,130	80.7	80.4–81.0	26,016	46.5	46.1–46.9
**Visa**					<0.001			<0.001			<0.001			<0.001
**Refugee**	74,025	70.2	67,685	91.4	91.2–91.6	70,656	95.5	95.3–95.6	56,341	76.1	75.8–76.4	35,923	48.5	48.2–48.9
**Special Immigrant Visa Holder**	11,801	11.2	10,221	86.6	86.0–87.2	11,015	93.3	92.9–93.8	9,806	83.1	82.4–83.8	7,233	61.3	60.4–62.2
**Asylee**	4,044	3.8	3,812	94.3	93.6–95.0	3,967	98.1	97.7–98.5	3,774	93.3	92.6–94.1	2,763	68.3	66.9–69.8
**Cuban/Haitian Entrant (Parolees)**	14,915	14.1	14,262	95.6	95.3–96.0	14,722	98.7	98.5–98.9	14,161	94.9	94.6–95.3	3,493	23.4	22.7–24.1
**Other (Nonrefugees)**^c^	666	0.6	626	94	92.2–95.8	644	96.7	95.3–98.1	605	90.8	88.7–93.0	371	55.7	51.9–59.5
**Nationality**					<0.001			<0.001			<0.001			<0.001
**Afghanistan**	11,736	11.1	10,203	86.9	86.3–87.6	10,982	93.6	93.3–94.0	9,586	81.7	81.0–82.4	7,163	61	60.2–61.9
**Bhutan**	4,221	4	3,839	91.0	90.1–91.8	3,984	94.4	93.7–95.1	2,752	65.2	63.8–66.6	1,851	43.9	42.4–45.4
**Burma**	17,749	16.8	15,839	89.2	88.8–89.7	16,786	94.6	94.2–94.9	13,732	77.4	76.8–78.0	8,604	48.5	47.7–49.2
**Democratic Republic of Congo**	6,554	6.2	5,909	90.2	89.4–90.9	6,315	96.4	95.9–96.8	4,937	75.3	74.3–76.4	2,799	42.7	41.5–43.9
**Cuba**	15,459	14.7	14,773	95.6	95.2–95.9	15,242	98.6	98.4–98.8	14,623	94.6	94.2–95.0	3,431	22.2	21.5–22.9
**Iran**	7,179	6.8	6,954	96.9	96.5–97.3	7,046	98.2	97.8–98.5	6,787	94.5	94.0–95.1	5,151	71.8	70.7–72.8
**Iraq**	16,605	15.8	15,062	90.7	90.3–91.2	16,057	96.7	96.4–97.0	13,241	79.7	79.1–80.4	8,229	49.6	48.8–50.3
**Somalia**	8,474	8	7,926	93.5	93.0–94.1	8,085	95.4	95.0–95.9	6,502	76.7	75.8–77.6	4,052	47.8	46.8–48.9
**Syria**	4,258	4	3,793	89.1	88.1–90.0	3,985	93.6	92.9–94.3	2,935	68.9	67.5–70.3	1,767	41.5	40.0–43.0
**Ukraine**	1,485	1.4	1,398	94.1	93.0–95.3	1,365	91.9	90.5–93.3	1,153	77.6	75.5–79.8	1,012	68.2	65.8–70.5
**Other**	11,731	11.1	10,910	93.0	92.5–93.5	11,157	95.1	94.7–95.5	8,439	71.9	71.1–72.8	5,724	48.8	47.9–49.7
**Last Residence**					<0.001			<0.001			<0.001			<0.001
**Afghanistan**	8,475	8	7,365	86.9	86.3–87.7	7,905	93.3	92.8–93.8	6,931	81.8	81.0–82.6	5,555	65.6	64.6–66.6
**Austria**	5,173	4.9	5,035	97.4	97.0–97.8	5,069	98.1	97.7–98.4	4,950	95.8	95.2–96.3	3,813	73.8	72.5–74.9
**Cuba**	3,791	3.6	3,603	95.0	94.4–95.8	3,688	97.3	96.8–97.8	3,213	84.8	83.6–85.9	1,494	39.4	37.9–41.0
**Iraq**	8,781	8.3	7,761	88.4	87.8–89.2	8,460	96.4	96.0–96.8	7,446	84.8	84.1–85.6	4,176	47.6	46.5–48.6
**Jordan**	4,851	4.6	4,322	89.1	88.3–90.0	4,585	94.5	93.9–95.2	3,633	74.9	73.7–76.1	2,244	46.3	44.9–47.7
**Kenya**	4,361	4.1	4,043	92.7	92.0–93.5	4,086	93.7	93.0–94.4	3,151	72.3	70.9–73.4	1,954	44.8	43.3–46.3
**Malaysia**	11,217	10.6	9,703	86.5	85.9–87.2	10,651	95	94.6–95.7	8,991	80.2	79.4–80.9	5,002	44.6	43.7–45.5
**Nepal**	4,319	4.1	3,917	90.7	90.2–91.9	4,038	93.5	92.8–94.3	2,843	65.8	64.4–67.3	2,001	46.3	44.8–47.8
**Thailand**	6,363	6	5,941	93.4	93.0–94.2	5,869	92.2	91.6–92.9	4,543	71.4	70.3–72.5	3,561	56	54.7–57.2
**Turkey**	5,586	5.3	5,209	93.3	92.7–94.0	5,416	97	96.5–97.4	4,653	83.3	82.3–84.3	3,348	59.9	58.7–61.2
**Other**	42,534	40.3	39,632	93.2	92.9–93.4	41,227	96.9	96.8–97.1	34,324	80.7	80.3–81.1	16,632	39.1	38.6–39.6
**Latent Tuberculosis Infection Test Type**														
**Interferon Gamma Release Assay**	85,972	81.5												
**Tuberculin Skin Test**	10,540	10												
**Not Done**	8,939	8.5												

Col% refers to proportion of persons with characteristics using the total population (in first column) as denominator, and Row% refers to proportion of persons screened using stratum-specific (row) total as denominator

^a^Excludes 10 individuals missing age.

^b^Excludes 20 individuals missing sex.

^c^“Other” visa category includes victims of trafficking, unaccompanied minors, and persons with an unspecified visa status.

Abbreviations: CI, confidence interval; HIV, human immunodeficiency virus

**Table 4:** Screening of refugees and other eligible immigrants for parasitic infections by demographic characteristics during domestic medical screening examinations conducted in eight^a^ US sites, 2014–2016.

Characteristics			Number and Proportion of Persons Screened											
	Total		Malaria			*Strongyloides*			*Schistosoma*			Other		
												Intestinal Parasites		
	*N*	Col%	*N*	Row%	χ^2^ 95% CI	*N*	Row%	χ^2^ 95% CI	*N*	Row%	χ^2^ 95% CI	*N*	Row%	χ^2^ 95% CI
**Total**	100,677	100.0	16,180	16.1	15.8–16.3	23,964	23.8	23.5–24.1	11,155	11.1	10.9–11.3	56,504	56.1	55.8–56.4
**Age (Years)**^b^					<0.001			<0.001			<0.001			<0.001
**<5**	11,417	11.3	1,489	13.0	12.4–13.7	3,550	31.1	30.2–31.9	1,397	12.2	11.6–12.8	6,658	58.3	57.4–59.2
**5**–**17**	23,492	23.3	3,591	15.3	14.8–15.7	6,043	25.7	25.2, 26.3	3,137	13.4	12.9–13.8	12,343	52.5	51.9–53.2
**18**–**44**	51,790	51.4	7,910	15.3	15.0–15.6	11,444	22.1	21.7–22.5	5,232	10.1	9.8–10.4	28,808	55.6	55.2–56.1
**45**–**64**	11,298	11.2	2,347	20.8	20.0–21.5	2,332	20.6	19.9–21.4	1,182	10.5	9.9–11.0	6,886	61.0	60.1–61.9
**65+**	2,670	2.7	840	31.5	29.7–33.2	586	22.0	20.4–23.5	207	7.8	6.7–8.8	1,800	67.4	65.6–69.2
**Examination Year**					<0.001			<0.001			<0.001			<0.001
**2014**	32,742	32.5	7,831	23.9	23.5–24.4	7,299	22.3	21.8–22.7	3,679	11.2	10.9–11.6	19,493	59.5	59.0–60.1
**2015**	31,924	31.7	7,057	22.1	21.7–22.6	6,954	21.8	21.3–22.2	3,697	11.6	11.2–11.9	17,410	54.5	54.0–55.1
**2016**	36,011	35.8	1,292	3.6	3.4–3.8	9,711	27.0	26.5–27.4	3,779	10.5	10.2–10.8	19,601	54.4	53.9–55.0
**Sex**^c^					<0.001			<0.001			0.1			0.3
**Female**	47,207	46.9	7,827	16.6	16.3–16.9	11,576	24.5	24.1–24.9	5,326	11.3	11.0–11.6	26,455	56.0	55.6–56.5
**Male**	53,450	53.1	8,353	15.6	15.3–15.9	12,387	23.2	22.8–23.5	5,828	10.9	10.6–11.2	30,047	56.2	55.8–56.6
**Visa**					<0.001			<0.001			<0.001			<0.001
**Refugee**	69,923	69.5	10,849	15.5	15.2–15.8	15,622	22.3	22.0–22.7	8,441	12.1	11.8–12.3	35,110	50.2	49.8–50.6
**Special Immigrant Visa Holder**	11,427	11.4	2,834	24.8	24.0–25.6	5,236	45.8	44.9–46.7	249	2.2	1.9–2.5	8,152	71.3	70.5–72.2
**Asylee**	3,910	3.9	2,009	51.4	49.8–53.0	773	19.8	18.5–21.0	184	4.7	4.0–5.4	3,202	81.9	80.7–83.1
**Cuban/Haitian Entrant (Parolees)**	14,775	14.7	289	2.0	1.7–2.2	2,070	14.0	13.5–14.6	2,172	14.7	14.1–15.3	9,584	64.9	64.1–65.6
**Other (Nonrefugees)**^d^	642	0.6	199	31.0	27.4–34.6	263	41.0	37.2–44.8	109	17.0	14.1–19.9	456	71.0	67.5–74.5
**Nationality**					<0.001			<0.001			<0.001			<0.001
**Afghanistan**	11,400	11.3	3,027	26.6	25.7–27.4	5,242	46.0	45.1–46.9	282	2.5	2.2–2.8	8,434	74.0	73.2–74.8
**Bhutan**	3,781	3.8	65	1.7	1.3–2.1	433	11.5	10.4–12.5	481	12.7	11.7–13.8	1,344	35.6	34.0–37.1
**Burma**	16,402	16.3	593	3.6	3.3–3.9	3,226	19.7	19.1–20.3	2,477	15.1	14.6–15.7	6,139	37.4	36.7–38.2
**Democratic Republic of Congo**	6,218	6.2	551	8.9	8.1–9.5	1,066	17.1	16.2–18.1	1,056	17.0	16.1–17.9	2,005	32.3	31.1–33.4
**Cuba**	15,300	15.2	282	1.8	1.6–2.1	2,350	15.4	14.8–15.9	2,353	15.4	14.8–16.0	9,892	64.7	63.9–65.4
**Iran**	7,141	7.1	3,488	48.8	47.7–50.0	1,703	23.9	22.9–24.8	58	0.8	0.6–1.0	6,360	89.1	88.3–89.8
**Iraq**	15,970	15.9	3,836	24.0	23.4–24.7	2,568	16.1	15.5–16.7	890	5.6	5.2–5.9	8,402	52.6	51.8–53.4
**Somalia**	7,928	7.9	887	11.2	10.5–11.9	3,034	38.3	37.2–39.3	2,199	27.7	26.8–28.7	3,481	43.9	42.8–45.0
**Syria**	4,171	4.1	216	5.2	4.5–5.9	1,014	24.3	23.0–25.6	209	5.0	4.4–5.7	2,208	52.9	51.4–54.5
**Ukraine**	1,403	1.4	335	23.9	21.7–26.1	936	66.7	64.3–69.2	74	5.3	4.1–6.4	1,038	74.0	71.7–76.3
**Other**	10,963	10.9	2,900	26.5	25.6–27.3	2,392	21.8	21.1–22.6	1,076	9.8	9.3–10.4	7,201	65.7	64.8–66.6
**Last Residence**					<0.001			<0.001			<0.001			<0.001
**Afghanistan**	8,170	8.1	2,504	30.6	29.6–31.6	4,367	53.5	52.4–54.5	212	2.6	2.3–2.9	6,397	78.3	77.4–79.2
**Austria**	5,173	5.1	2,715	52.5	51.1–53.8	1,127	21.8	20.7–22.9	36	0.7	0.5–0.9	4,792	92.6	91.9–93.4
**Cuba**	3,642	3.6	126	3.5	2.9–4.1	1,831	50.3	48.7–51.9	1,849	50.8	49.1–52.4	2,668	73.3	71.8–74.7
**Iraq**	8,333	8.3	1,715	20.6	19.7–21.5	1,335	16.0	15.2–16.8	461	5.5	5.0–6.0	3,716	44.6	43.5–45.7
**Jordan**	4,727	4.7	663	14.0	13.0–15.0	1,082	22.9	21.7–24.1	291	6.2	5.5–6.8	2,708	57.3	55.9–58.7
**Kenya**	3,989	4.0	411	10.3	9.4–11.3	1,090	27.3	25.9–28.7	877	22.0	20.7–23.3	1,506	37.8	36.3–39.3
**Malaysia**	10,283	10.2	458	4.5	4.1–4.9	1,528	14.9	14.2–15.6	332	3.2	2.9–3.6	3,476	33.8	32.9–34.7
**Nepal**	3,859	3.8	141	3.7	3.1–4.3	479	12.4	11.4–13.5	494	12.8	11.8–13.9	1,290	33.4	31.9–34.9
**Thailand**	5,946	5.9	277	4.7	4.1–5.2	1,639	27.6	26.4–28.7	2,118	35.6	34.4–36.8	2,282	38.4	37.1–39.6
**Turkey**	5,444	5.4	1,645	30.2	29.0–31.4	1,457	26.8	25.6–27.9	331	6.1	5.5–6.7	3,817	70.1	68.9–71.3
**Other**	41,111	40.8	5,525	13.4	13.1–13.8	8,029	19.5	19.2–19.9	4,154	10.1	9.8–10.4	23,852	58.0	57.5–58.5

Col% refers to proportion of persons with characteristics using the total population (in first column) as denominator, and Row% refers to proportion of persons screened using stratum-specific (row) total as denominator.

^a^Excludes data from one site that did not collect information on total numbers of persons screened for these conditions.

^b^Excludes 10 individuals missing age.

^c^Excludes 20 individuals missing sex.

^d^“Other” visa category includes victims of trafficking, unaccompanied minors, and persons with an unspecified visa status.

Abbreviation: CI, confidence interval

**Table 5:** Screening for sexually transmitted infections of refugees and other eligible immigrants by demographic characteristics during domestic medical screening examinations conducted in eight^a^ US sites, 2014–2016.

Characteristics	Number and Proportion of Persons Screened										
	Total		Syphilis			Chlamydia			Gonorrhea		
	*N*	Col%	*N*	Row%	χ^2^ 95% CI	*N*	Row%	χ^2^ 95% CI	*N*	Row%	χ^2^ 95% CI
**Total**	100,677	100	60,713	60.3	60.0–60.6	14,102	14	13.8–14.2	4,687	4.7	4.5–4.8
**Age (Years)**^b^					<0.001			<0.001			<0.001
**<5**	11,417	11.3	1,045	9.2	8.6–9.7	75	0.7	0.5–0.8	10	0.1	0.03–0.1
**5**–**17**	23,492	23.3	6,705	28.5	28.0–29.1	2,206	9.4	9.0–9.8	753	3.2	3.0–3.4
**18**–**44**	51,790	51.4	41,512	80.2	79.8–80.5	10,214	19.7	19.4–20.1	3,226	6.2	6.0–6.4
**45**–**64**	11,298	11.2	9,230	81.7	81.0–82.4	1,340	11.9	11.3–12.5	573	5.1	4.7–5.5
**65+**	2,670	2.7	2,212	82.9	81.4–84.3	259	9.7	8.6–10.8	125	4.7	3.9–5.5
**Exam Year**					<0.001			<0.001			<0.001
**2014**	32,742	32.5	19,714	60.2	59.7–60.7	3,047	9.3	9.0–9.6	1,159	3.5	3.3–3.7
**2015**	31,924	31.7	20,015	62.7	62.2–63.2	4,952	15.5	15.1–15.9	1,490	4.7	4.4–4.9
**2016**	36,011	35.8	20,984	58.3	57.8–58.8	6,103	17	16.6–17.3	2,038	5.7	5.4–5.9
**Sex**^c^					<0.001			<0.001			<0.001
**Female**	47,207	46.9	27,999	59.3	58.9–59.8	8,913	18.9	18.5–19.2	2,459	5.2	5.0–5.4
**Male**	53,450	53.1	32,712	61.2	60.8–61.6	5,189	9.7	9.5–10.0	2,228	4.2	4.0–4.3
**Visa**					<0.001			<0.001			<0.001
**Refugee**	69,923	69.5	37,224	53.2	52.9–53.6	10,174	14	14.3–14.8	4,401	6.3	6.1–6.5
**Special Immigrant Visa Holder**	11,427	11.4	6,634	58.1	57.2–59.0	1,886	16.5	15.8–17.2	25	0.2	0.1–0.3
**Asylee**	3,910	3.9	3,134	80.2	78.9–81.4	791	20.2	19.0–21.5	10	0.3	0.1–0.4
**Cuban/Haitian Entrant (Parolees)**	14,775	14.7	13,283	89.9	89.4–90.4	1,111	7.52	7.1–7.9	192	1.3	1.1–1.5
**Other (Nonrefugees)**^d^	642	0.6	438	68.2	64.6–71.8	140	21.8	18.6–25.0	59	9.2	7.0–11.4
**Nationality**					<0.001			<0.001			<0.001
**Afghanistan**	11,400	11.32	6,545	57.4	56.5–58.3	2,172	19.1	18.3–19.8	62	0.5	0.4–0.7
**Bhutan**	3,781	3.76	1,980	52.4	50.8–54.0	624	16.5	15.3–17.7	293	7.8	6.9–8.6
**Burma**	16,402	16.3	6,903	42.1	41.3–42.8	2,049	12.5	12.0–13.0	1,371	8.4	7.9–8.8
**Democratic Republic of Congo**	6,218	6.2	3,032	48.8	47.5–50.0	919	14.8	13.9–15.7	369	5.9	5.4–6.5
**Cuba**	15,300	15.2	13,642	89.2	88.7–89.7	1,155	7.6	7.1–8.0	231	1.5	1.3–1.7
**Iran**	7,141	7.1	6,197	86.8	85.7–87.6	1,019	14.3	13.5–15.1	42	0.6	0.4–0.8
**Iraq**	15,970	15.9	9,883	61.9	61.1–62.6	1,659	10.4	9.9–10.9	373	2.3	2.1–2.6
**Somalia**	7,928	7.9	3,730	47.1	46.0–48.2	1,686	21.3	20.4–22.2	1,304	16.5	15.6–17.3
**Syria**	4,171	4.1	1,737	41.6	40.2–43.1	451	10.8	9.9–11.8	102	2.5	2.0–2.9
**Ukraine**	1,403	1.4	838	59.7	57.2–62.3	453	32.3	29.8–34.7	69	4.9	3.8–6.1
**Other**	10,963	10.9	6,226	56.8	55.9–57.7	1,915	17.5	16.8–19.2	471	4.3	3.9–4.7
**Last Residence**					<0.001			<0.001			<0.001
**Afghanistan**	8,170	8.1	4,550	55.7	54.6–56.8	1,648	20.2	19.3–21.0	40	0.5	0.3–0.6
**Austria**	5,173	5.1	4,635	89.6	88.8–90.4	621	12	11.1–12.9	31	0.6	0.4–0.8
**Cuba**	3,642	3.6	2,842	78	76.7–79.4	451	12.4	11.3–13.5	197	5.4	4.7–6.1
**Iraq**	8,333	8.3	5,342	64.1	63.1–65.1	835	10	9.4–10.7	218	2.6	2.3–3.0
**Jordan**	4,727	4.7	2,350	49.7	48.3–51.1	578	12.2	11.3–13.2	144	3.1	2.6–3.5
**Kenya**	3,989	4	1,868	46.8	45.3–48.4	801	20.1	18.8–21.3	545	13.7	12.6–14.7
**Malaysia**	10,283	10.2	4,429	43.1	42.1–44.0	841	8.2	7.7–8.7	436	4.2	3.4–4.6
**Nepal**	3,859	3.8	1,987	51.5	49.9–53.1	727	18.8	17.6–20.1	318	8.2	7.4–9.1
**Thailand**	5,946	5.9	2,464	41.4	40.2–42.7	1,241	20.9	19.8–21.9	899	15.1	14.2–16.0
**Turkey**	5,444	5.4	3,651	67.1	65.8–68.3	910	16.7	15.7–17.7	133	2.4	2.0–2.9
**Other**	41,111	40.8	26,595	64.7	64.2–65.2	5,449	13.3	12.9–13.6	1,726	4.2	4.0–4.4

Col% refers to proportion of persons with characteristics using the total population (in first column) as denominator, and Row% refers to proportion of persons screened using stratum-specific (row) total as denominator.

^a^Excludes data from one site that did not collect information on total numbers of persons screened for these conditions.

^b^Excludes 10 individuals missing age.

^c^Excludes 20 individuals missing sex.

^d^“Other” visa category includes victims of trafficking, unaccompanied minors, and persons with an unspecified visa status.

Abbreviation: CI, confidence interval

**Table 6:** Screening for blood lead levels and mental health of refugees and other eligible immigrants by demographic characteristics during domestic medical screening examinations conducted in multiple sites, 2014–2016.

Characteristics	Number and Proportion of Persons Screened									
	Total^a^		Lead			Total^b^		Mental Health		
	*N*	Col%	*N*	Row%	χ^2^ 95% CI	*N*	Col%	*N*	Row%	χ^2^ 95% CI
**Total**	35,118	100	31,108	88.6	88.3–88.9	27,712	100	10,208	36.8	36.3–37.4
**Age (Years)**^c^					<0.001					<0.001
**≤2**	6,875	19.6	5,635	82.0	81.1–82.9	1,970	7.1	18	0.9	0.5–1.3
**3–6**	9,741	27.7	8,867	91.0	90.5–91.6	2,754	9.9	28	1.0	0.6–1.4
**7–12**	11,836	33.7	10,809	91.3	90.8–91.8	3,457	12.5	37	1.1	0.7–1.4
**13–16**	6,656	19.0	5,796	87.1	86.3–87.9	2,015	7.3	455	22.6	20.8–24.4
**17–44**						14,386	51.9	7,861	54.6	53.8–55.5
**45–64**						2,680	9.7	1,580	59	57.1–60.8
**65+**						449	1.6	228	50.8	46.2–55.4
**Exam Year**					<0.001					<0.001
**2014**	10,761	30.6	9,409	87.4	86.8–88.1	8,940	32.3	2,943	32.9	32.0–33.9
**2015**	10,722	30.5	9,479	88.4	87.8–89.0	8,226	29.7	3,122	38	36.9–39.0
**2016**	13,635	38.8	12,220	89.6	89.1–90.1	10,546	38.1	4,143	39.3	38.4–40.2
**Sex**^d^					0.03					0.02
**Female**	17,007	48.4	15,004	88.2	87.7–88.7	13,232	47.7	4,781	36.1	35.3–37.0
**Male**	18,103	51.5	16,102	89	88.5–89.4	14,466	52.2	5,426	37.5	36.7–38.3
**Visa Type**					<0.001					<0.001
**Refugee**	27,566	78.5	24,757	89.8	89.5–90.2	23,351	84.3	7,427	31.8	31.2–32.4
**Special Immigrant Visa Holder**	4,998	14.2	4,270	85.4	84.5–86.4	1,128	4.1	347	30.8	28.1–33.5
**Asylee**	1,083	3.1	845	78	75.6–80.5	197	0.7	125	63.5	56.7–70.2
**Cuban/Haitian Entrant (Parolees)**	1,269	3.6	1,067	84.1	82.1–86.1	2,956	10.7	2,257	76.4	74.8–77.9
**Other (Nonrefugees)**^e^	202	0.6	169	83.7	78.6–88.8	80	0.3	52	65.0	54.6–75.5
**Nationality**					<0.001					<0.001
**Afghanistan**	4,986	14.2	4,175	83.7	82.7–84.8	1,249	4.5	360	28.8	26.3–31.3
**Bhutan**	1,194	3.4	1,098	92	90.4–93.5	2,266	8.2	790	34.9	32.9–36.8
**Burma**	6,675	19	5,982	89.6	88.9–90.4	7,654	27.6	2,349	30.7	29.7–31.7
**Democratic Republic of Congo**	3,223	9.2	3,025	93.9	93.0–94.7	3,132	11.3	994	31.7	30.1–33.4
**Cuba**	1,395	4.0	1,196	85.7	83.9–87.6	3,287	11.9	2,481	75.5	74.0–77.0
**Iran**	934	2.7	697	74.6	71.8–77.4	100	0.4	51	51.0	41.2–60.8
**Iraq**	5,515	15.7	4,852	88	87.1–88.8	2,708	9.8	1,089	40.2	38.4–42.1
**Somalia**	3,795	10.8	3,501	92.3	91.4–93.1	3,026	10.9	844	27.9	26.3–29.5
**Syria**	2,252	6.4	2,033	90.3	89.1–91.5	1,201	4.3	374	31.1	28.5–33.8
**Ukraine**	536	1.5	442	82.5	79.2–85.7	297	1.1	75	25.3	20.3–30.2
**Other**	4,613	13.1	4,107	89	88.1–89.9	2,792	10.1	801	28.7	27.0–30.4
**Last Residence**					<0.001					<0.001
**Afghanistan**	3,809	10.8	3,163	83	81.9–84.2	891	3.2	265	29.7	26.7–32.7
**Austria**	577	1.6	398	69	65.2–75.8	9	0	1	11.1	0–48.3*
**Cuba**	505	1.4	452	89.5	86.8–92.2	2,598	9.4	1,948	75	73.3–76.7
**Iraq**	3,084	8.8	2,729	88.5	87.4–89.6	1,575	5.7	673	42.7	40.3–45.2
**Jordan**	2,236	6.4	2,006	98.7	88.5–91.0	1,238	4.5	408	33.0	30.3–35.6
**Kenya**	1,960	5.6	1,802	91.9	90.7–93.1	1,846	6.7	482	26.1	24.1–28.1
**Malaysia**	4,027	11.5	3,561	88.4	87.4–89.4	5,337	19.3	1,833	34.4	33.1–35.6
**Nepal**	1,295	3.7	1,187	91.7	90.2–93.2	2,441	8.8	811	33.2	31.4–35.1
**Thailand**	2,768	7.9	2,768	93	92.0–93.9	2,362	8.5	482	20.4	18.8–22.0
**Turkey**	1,764	5.0	1,764	88.2	86.6–89.7	899	3.2	326	36.3	33.1–39.4
**Other**	13,093	37.3	11,681	89.2	88.7–89.8	8,516	30.7	2,979	35.0	34.0–36.0

Col% refers to proportion of persons with characteristics using the total population (in first column) as denominator, and Row% refers to proportion of persons screened using stratum-specific (row) total as denominator.

^a^Total for lead screening only, excludes persons ≥17 years old.

^b^Total for mental health screening only, excludes persons from four sites not reporting information on mental health screening.

^c^Excludes 10 individuals missing age.

^d^Excludes two records with unknown sex for lead, one with unknown sex for mental health.

^e^“Other” visa category includes victims of trafficking, unaccompanied minors, and persons with an unspecified visa status.

Abbreviation: CI, confidence interval

Approximately 51% of persons in our initial data set were between 18 and 44 years old, and 34.8% were under age 18 (Table 2). Fifty-three percent were male. Burma (16.8%), Iraq (15.8%), Cuba (14.7%), Afghanistan (11.1%), and Somalia (8.0%) contributed the largest volumes of arrivals by nationality (Table 2). The data set mostly comprised refugees (70.2%), Cuban/Haitian parolees (14.1%), SIVHs (11.2%), and asylees (3.8%) (Table 2). Fifty percent of examinations took place within 30 days of US arrival (Table 2). Timing of screening after arrival varied by visa type (refugees and SIVH: median 27 days; parolees: 49 days; asylees: 57 days, Table 2), consistent with anticipated flows through the resettlement process.

Most individuals (96,606 of 105,541; 91.6%, 95% CI 91.4–91.8) were screened for TB (Table 3); of those, 89.0% were screened with an IGRA test. Screening differed significantly by age, with 95.5% (95% CI 95.3–95.6, collapsed from Table 3) of those aged 6 years or older screened for TB compared with 61.9% (95% CI 61.1–62.8) of children aged 5 and younger (χ^2^ *p* < 0.001). Screening was lowest among SIVH (86.6%, 95% CI 86.0–87.2) compared with all visa types (91.6%, 95% CI 91.4–91.8). Afghans had the lowest proportion of persons screened for TB (86.9%, 95% CI 86.3–87.6); Iranians had the highest (96.9%, 95% CI 96.5–97.3).

In our data set, hepatitis B virus (HBV) screening was reported for 101,004 of 105,541 persons (95.8%, 95% CI 95.7–95.9) (Table 3). Screening coverage among children <5 years old was lowest (86.0%, 95% CI 85.4–86.6); ≥98.3% of persons ≥45 years old were screened. A lower proportion of SIVHs were screened (93.3%, 95% CI 92.9–93.8) compared with parolees and asylees (≥98.1%). Screening coverage was lowest among Ukrainians (91.9%, 95% CI, 90.5–93.3) and highest among Cubans (98.6%, 95% CI 98.4–98.8).

HIV screening was reported for 84,687 of 105,541 persons (80.3%, 95% CI 80.1–80.6) (Table 3). Screening coverage differed significantly across age groups (χ^2^ *p* < 0.001), with the lowest coverage among children less than 5 years old (65.0%, 95% CI 64.2–65.9). The percentage of adults ≥18 years old screened for HIV was 85.2% (95% CI 84. 9–85.4, data collapsed from Table 3). Among top arrival nationalities, screening ranged from 65.2% (95% CI 63.8–66.6) among Bhutanese to 94.6% (95% CI 94.2–95.0) among Cubans.

Hepatitis C virus (HCV) screening was reported for 49,783 (47.2%, 95% CI 46.9–47.5) of 105,541 persons (Table 3). Screening coverage increased with age; 56.1% (95% CI 55.3–56.9, data collapsed from Table 3) of adults 45 years and older at the time of their examination (ages corresponding loosely to persons born between 1945 and 1965, a population with greater HCV infection risk in the US) were tested for HCV; screening coverage among person <45 years old was 45.8% (95% CI 45.5–46.1, data collapsed from Table 3). Asylees had the highest screening coverage (68.3%, 95% CI 66.9–69.8). The screening coverage of persons from countries with high or high-moderate HCV burden [15] included 68.2% (95% CI 65.8–70.5) of Ukrainians, 42.7% (95% CI 41.5–43.9) of Congolese (Democratic Republic), and 41.5% (95% CI 40.0–43.0) of Syrians.

Our data set consisted of 100,677 persons with data on screening coverage for malaria, *Strongyloides*, *Schistosoma*, and other intestinal parasites. In this data set, 16,180 (16.1%, 95% CI 15.8–16.3) persons were reported screened for malaria (Table 4). Asylees had the highest screening coverage (51.4%, 95% CI 49.8–53.0). Screening declined from 23.9% (95% CI 23.5–24.4) of persons receiving a domestic medical examination in 2014 to 3.6% (95% CI 3.4–3.8) of persons arriving in 2016. Nationalities with the highest proportion of persons screened included Iranians (48.8%, 95% CI 47.7–50.0) and Afghans (26.6%, 95% CI 25.7–27.4). People with a last residence in Austria, Afghanistan, or Turkey had the highest screening percentage.

Of the 100,677 persons screened for intestinal parasites, 23,964 (23.8%, 95% CI 23.5–24.1) were screened for *Strongyloides* infection (Table 4). Ukrainians (66.7%, 95% CI 64.3–69.2), Afghans (46.0%, 95% CI 45.1–46.9), and Somalis (38.3%, 95% CI 37.2–39.3) had the highest proportion of persons screened for *Strongyloides*. Screening was highest among persons last residing in Afghanistan (53.5%, 95% CI 52.4–54.5) or Cuba (50.3%, 95% CI 48.7–51.9). *Schistosoma* infection screening was reported for 11,155 of 100,677 (11.1%, 95% CI 10.9–11.3) persons; screening was highest among persons 5–17 years old (13.4%, 95% CI 12.9–13.8). Parolees (14.7%, 95% CI 14.1–15.3) and refugees (12.1%, 95% CI 11.8–12.3), Somalis (27.7%, 95% CI 26.8–28.7), and persons last residing in Cuba (50.8%, 95% CI 49.1–52.4) had the highest proportions of persons screened.

Approximately 56% (95% CI 55.8–56.4) of 100,677 persons were screened for other intestinal parasites, such as *Ascaris* (Table 4). Screening coverage was highest among asylees (81.9%, 95% CI 80.7–83.1), SIVHs (71.3%, 95% CI 70.5–72.2) and parolees (64.9%, 95% CI 64.1–65.6), Iranians (89.1%, 95% CI 88.3–89.8), and Afghans (74.0%, 95% CI 73.2, 74.8). Screening was highest among persons with a last residence in Austria (92.6%, 95% CI 91.9–93.4), Afghanistan (78.3%, 95% CI 77.4–79.2), and Cuba (73.3%, 95% CI 71.8–74.7).

Among 100,677 persons with screening data for STIs, 60,713 (60.3%, 95% CI 60.0–60.6) were screened for syphilis (Table 5). Proportions of persons screening differed significantly by age (χ^2^ *p* < 0.001). Screening coverage was lowest among children younger than 5 years (9.2%, 95% CI 8.6–9.7) and 5–17 years (28.5%, 95% CI 28.0–29.1) compared with persons 18 years and older (80.5%, 95% CI 80.2–80.8, data collapsed from Table 5). Screening was highest among parolees (89.9%, 95% CI 89.4–90.4) and asylees (80.2%, 95% CI 78.9–81.4).

A smaller proportion of our data set were screened for chlamydia (14,102 of 100,677 persons, 14.0%, 95% CI 13.8–14.2). Chlamydia screening was highest among persons aged 18–44 years, SIVHs and asylees, women, and Ukrainians. Gonorrhea screening results were available for 4,687 of 100,677 persons (4.7%, 95% CI 4.5–4.8). Screening was highest among persons 18–44 years, refugees, and Somalis.

The majority (31,108 of 35,118; 88.6%, 95% CI 88.3–88.9) of children <17 years of age had blood lead screening results (Table 6). Screening coverage was significantly lower among children ≤2 years old (82.0%, 95% CI 81.1–82.9) compared with children ≥3 years old (90.2%, 95% CI 89.9–90.6, data collapsed from Table 6). Screening was highest among refugees (89.8%, 95% CI 89.5–90.2) and lowest among asylees (78.0%, 95% CI 75.6–80.5) and was lower among children with a last residence in Austria (69.0%, 95% CI 65.2–75.8) compared with other countries of last residence.

Among the five sites that provided mental health screening data (denominator: 27,712 persons), screening was reported for 10,208 (36.8%, 95% CI 36.3–37.4) persons (Table 6). Mental health screening was low among children <16 years old (3.9% 95% CI 3.5–4.3, data collapsed from Table 6); 16 is the age at which mental health screening is recommended in CDC’s *Guidelines for the U*.*S*. *Domestic Medical Examination for Newly Arriving Refugees*. Screening among persons ≥16 years old was 54.6% (95% CI 53.9–55.3, data collapsed from Table 6). The proportion of persons who received mental health screening increased slightly between 2014 and 2016, and screening proportion differed by examination year (χ^2^ *p* < 0.001). A higher proportion of asylees (63.5%, 95% CI 56.7–70.2) and parolees (76.4%, 95% CI 74.8–77.9) received mental health screening than refugees (31.8, 95% CI 31.2–32.4) and SIVHs (30.8%, 95% CI 28.1–33.5).

## Discussion

This analysis used a multistate data set to describe disease screenings conducted during the domestic medical examination for refugees and other eligible populations. These results provide new evidence that most persons eligible for refugee health benefits are screened for several health conditions after US arrival through the domestic medical examination, particularly for TB (91.6%), HBV (95.8%), HIV (80.3%), and blood lead (88.6% of persons <17 years old). Screening coverage for other health conditions, including STIs, parasites, and mental health, was lower. For most conditions, screening coverage increased with age and differed by nationality and country of last residence. Differences in proportions screened by sex and year of health assessment, although statistically significant, were not of clinical or public health significance for most conditions.

To our knowledge, this is the first analysis of a large, multisite data set to assess screening coverage resulting from the domestic medical examination recommended for newly arrived refugees and other eligible visa holders in the US. Our data are consistent with prior studies that have reported high screening coverage for TB (93%) [16] and HBV (92.5%) during the domestic medical examination [11,13,16]. Compared with two studies reporting screening coverage for intestinal parasites among refugees in Minnesota (64%–80%) [16,17], our analysis found a lower screening coverage among refugees (50.2%). However, the Minnesota studies predate the current overseas presumptive parasite treatment program for US-bound refugees. Now, screening for intestinal parasites may be considered based on clinical symptoms and results of diagnostic testing (e.g., eosinophilia). Reports on screening coverage during the domestic medical examination for other conditions were lacking for comparison.

Our findings indicate that most persons undergoing the domestic medical examination receive several routine health screenings recommended in the US, resulting in higher screening coverage in this population compared with the broader US population for several conditions. In the US, TB and HBV screening are recommended for populations at highest risk of infections [18,19], including foreign-born persons from countries with moderate to high burden of the diseases [20,21]. In our analysis, 91.6% of persons who received a domestic medical examination were screened for TB compared with roughly 63% of all foreign-born respondents in the 2011–2012 National Health and Nutritional Examination Survey (self-reported data representative of persons residing in the US) [22]. Almost 96% of persons in our data set were screened for HBV compared with 19% of patients enrolled in four US healthcare organizations regardless of risk factors (*N* = 1,248,558) [23]. CDC recommends screening all adults for HIV at least once in their lifetime [24]. We found that 85% of adults were tested for HIV during the domestic screening examination, which is higher than the testing rate among the US adult population (<40% in 2016–2017) [24]. Finally, during the period of this analysis, one-time HCV screening was recommended for all US adults born between 1945 and 1965, regardless of risk factors [25,26]. The HCV screening rate among persons in our data set falling into this birth cohort (>44 years old at the time of their examination) was 56.1% (95% CI 55.3–56.9, data collapsed from Table 3), which is higher than the HCV screening rate among the general US population born between 1945 through 1965 (13% ever screened in 2015) [27]. The relatively high screening rates for TB, HBV, HIV, and HCV during the domestic examination demonstrates that the domestic examination is an effective opportunity to identify eligible new arrivals with these conditions and link them to care in the US. However, efforts to explore why screening opportunities for these conditions are missed during the domestic medical examination may further improve screening coverage.

Screening coverage for malaria and intestinal parasites varied widely by visa type, nationality, and country of last residence. Screening coverage for malaria and intestinal parasites was lower among refugees than among other visa holders, which is likely the result of the overseas presumptive parasite treatment; if complete overseas treatment is documented, domestic screening is not required [28]. Ukrainians are likely overscreened for *Strongyloides* infection based on unclear guidance for screening for refugees originating from Europe, where *Strongyloides* is not hyperendemic [29]. Domestic screening is still recommended for persons who are symptomatic or have signs of infection such as eosinophilia, those who were ineligible for overseas treatment because of contraindications, and those from countries where presumptive treatment is not available.

The CDC *Guidelines for the U*.*S*. *Domestic Medical Examination for Newly Arriving Refugees* recommend screening for certain STIs based on whether overseas screening was done (for syphilis), age, and other risk factors, including history of sexual assault [7]. The proportions of refugees and SIVH screened for syphilis (53% and 58%, respectively) were lower compared with other visa types, likely reflecting the impact of overseas immigration screening requirements for syphilis. Domestic clinicians are recommended to review overseas syphilis testing results and to repeat testing in the US if overseas results are unavailable. For most of the period of this analysis, gonorrhea testing was not required overseas (overseas testing began October 1, 2016), and chlamydia testing has never been required overseas. For these infections, the domestic guidelines recommend screening for patients with certain risk factors (age, sexual history, or symptoms). Domestic gonorrhea and chlamydia screening among these populations could reflect presence of risk factors or symptoms, availability of overseas testing results, clinic screening policies for these conditions, or cultural barriers that discourage refugees from disclosing risk factors [30].

Blood lead screening was reported for 88.6% of all children <17 years old; children ≤2 years old had the lowest screening rates (82.0%). CDC’s *Guidelines for the U*.*S*. *Domestic Medical Examination for Newly Arriving Refugees* recommend screening children aged 6 months through 16 years for lead upon arrival, but our data did not permit us to uniformly identify children younger than 1 year. Consequently, 82.0% is likely an underestimate of the proportion of children appropriately screened in this youngest age group. However, children aged 1–2 still had lower screening coverage compared with older children. Prior investigations have found a high prevalence of elevated blood lead levels among refugees [12,31–33], and infants and young children are at greatest risk of both lead exposures and the resulting negative health outcomes [33]. Understanding and addressing barriers to lead screening among infants and young children is critical to ensuring appropriate care. Moreover, results from blood lead screening performed during the domestic medical exam serve as a baseline for the recommended repeat screening 3–6 months following resettlement that may highlight potential domestic exposures.

CDC’s *Guidelines for the U*.*S*. *Domestic Medical Examination for Newly Arriving Refugees* encourage screening for depression and posttraumatic stress disorders (PTSDs) among people over age 16. In our analysis, mental health screening coverage among adults was between 50% and 55% and was lowest among refugees (31.8%) and SIVHs (30.8%). The relatively low rate of mental health screening could be the result of multiple factors. First, immigration-related screening has historically prioritized infectious disease screening in order to reduce disease risks to refugees and their resettlement communities. Second, mental health screening may be deferred from the domestic medical examination, which is typically administered within the first 90 days of US arrival, because refugees may be in a period of adjustment in which mental health symptoms are not as apparent [34,35], or they may not feel comfortable disclosing symptoms during the initial examination [7]. After building a rapport with clinicians, both care providers and resettled refugees may be better positioned to screen for and address mental health issues. Finally, research has found that screening is often informally administered, which may have made it difficult to capture in our data source, and screening may be less likely to be administered if culturally and linguistically competent mental health services are unavailable in the screening jurisdiction for referrals [36,37]. However, we were not able to ascertain why mental health screening was not done in this analysis. Meta-analyses of mental health among refugees and other conflict-affected populations have estimated prevalence of depression (>23%) and PTSDs (also >23%) [38,39] that exceed the estimated prevalence of these conditions among other populations in the US, such as veterans (PTSD 8%) and the US household population (depression 7.6%). The high prevalence of mental health conditions coupled with the low screening coverage in our analysis highlights the need for increased investigation of when and how mental health screening is being conducted among refugees and other populations receiving the domestic medical examination, barriers to mental health screening and services, and effective treatments for these populations.

This analysis was subject to limitations. The data upon which we relied were often collected primarily for programmatic rather than surveillance purposes, which yielded gaps in information (e.g., screening test type). Sites used screening tests and algorithms that may differ in sensitivity and specificity to detect conditions, and they collected and shared data differently. Therefore, our findings only reflect those sites contributing data and not all refugee and other persons receiving the domestic medical screening examination in the US. Some sites did not routinely collect information on each health condition included in the analysis, but this may not mean that they did not screen for these conditions; conversely, it also is possible that sites reported testing done outside the domestic medical examination, depending on their source of data and method of data extraction. One site captured only positive screening outcomes for parasitic infections and STIs without a denominator of all persons tested; we excluded these results from our calculations. Screening for some conditions is based on patient age, region of origin, or other characteristics (e.g., blood lead, malaria, STIs), so not all patients should have received screening for each health condition. Screening is done at the discretion of the examining clinician, with some healthcare providers being more likely to screen for certain conditions than others; and all screening tests can be declined by the recipients, which may be influenced by sex, age, cultural factors, and type of testing. We were not able to capture information on why an individual may not have been screened. Finally, we were unable to collect data on linkage to care in this population and could not evaluate how well the domestic medical screening examination connected people to the US healthcare system for further care.

This analysis found that refugees and other recipients of the domestic screening examination are well screened for several health conditions shortly after arrival in the US. This indicates that the domestic medical examination is effective at its intended purpose to screen newly resettled persons for health conditions, protecting both their health and the health of the receiving community. Future directions for research could include exploration of screening practices during the domestic medical examination, particularly mental health screening, and assessment of the domestic examination’s effectiveness at connecting recipients to the US healthcare system.

Our assessment of a large, multisite data set found that screening coverage for several key health conditions—TB, hepatitis B and C, and HIV—was high during domestic screening examination, particularly when compared with screening rates among the US population. The domestic medical examination provides a good opportunity to improve the health of newly arrived refugees, SIVHs, asylees, and parolees by ensuring they receive several routine recommended health screenings.

## Supporting information

S1 STROBE Checklist STROBE checklist. STROBE, Strengthening the Reporting of Observational Studies in Epidemiology.
(DOCX)

## Abbreviations

anti-HBc: hepatitis B core antibody
anti-HBs: hepatitis B surface antibody
CDC: Centers for Disease Control and Prevention
CI: confidence interval
HBsAg: hepatitis B surface antigen
HBV: hepatitis B virus
HCV: hepatitis C virus
HIV: human immunodeficiency virus
IGRA: interferon gamma release assay
LTBI: latent TB infection
PCR: polymerase chain reaction
PTSD: posttraumatic stress disorder
RHS: Refugee Health Screener
RIBA: recombinant immunoblot assay
SD: standard deviation
SIVH: Special Immigrant Visa holder
STI: sexually transmitted infection
STROBE: Strengthening the Reporting of Observational Studies in Epidemiology
TB: tuberculosis
TST: tuberculin skin test
